# Developing Post-Consumer Recycled Flexible Polypropylene and Fumed Silica-Based Nanocomposites with Improved Processability and Thermal Stability

**DOI:** 10.3390/polym15051142

**Published:** 2023-02-24

**Authors:** Eliezer Velásquez, Cristian Patiño Vidal, Guillermo Copello, Carol López de Dicastillo, C. J. Pérez, Abel Guarda, María José Galotto

**Affiliations:** 1 Packaging Innovation Center (LABEN-Chile), University of Santiago of Chile (USACH), Santiago 9170201, Chile; 2 Center for the Development of Nanoscience and Nanotechnology (CEDENNA), University of Santiago of Chile, Santiago 9170124, Chile; 3 Departamento de Ciencias Químicas, Facultad de Farmacia y Bioquímica, Universidad de Buenos Aires, Buenos Aires C1113AAD, Argentina; 4 Instituto de Química y Metabolismo del Fármaco (IQUIMEFA), CONICET—Universidad de Buenos Aires, Buenos Aires C1113AAD, Argentina; 5 Packaging Laboratory, Institute of Agrochemistry and Food Technology (IATA-CSIC), 46980 Paterna, Spain; 6 Institute of Materials Science and Technology (INTEMA), National University of Mar del Plata-National Research Council (CONICET), Mar del Plata 7600, Argentina; 7 Food Science and Technology Department, Technological Faculty, University of Santiago of Chile (USACH), Santiago 9170201, Chile

**Keywords:** nanocomposite, post-consumer polypropylene, fumed silica, processability, extrusion

## Abstract

Collection and mechanical recycling of post-consumer flexible polypropylene packaging is limited, principally due to polypropylene being very light-weight. Moreover, service life and thermal–mechanical reprocessing degrade PP and change its thermal and rheological properties according to the structure and provenance of recycled PP. This work determined the effect of incorporating two fumed nanosilica (NS) types on processability improvement of post-consumer recycled flexible polypropylene (PCPP) through ATR-FTIR, TGA, DSC, MFI and rheological analysis. Presence of trace polyethylene in the collected PCPP increased the thermal stability of the PP and was significantly maximized by NS addition. The onset decomposition temperature raised around 15 °C when 4 and 2 wt% of a non-treated and organically modified NS were used, respectively. NS acted as a nucleating agent and increased the crystallinity of the polymer, but the crystallization and melting temperatures were not affected. The processability of the nanocomposites was improved, observed as an increase in viscosity, storage and loss moduli with respect to the control PCPP, which were deteriorated due to chain scission during recycling. The highest recovery in viscosity and reduction in MFI were found for the hydrophilic NS due to a greater impact of hydrogen bond interactions between the silanol groups of this NS and the oxidized groups of the PCPP.

## 1. Introduction

In recent years, considering that global plastics production increased to 390.7 million tons in 2021, development of strategies for valuing plastic waste has received growing interest in order to reduce their accumulation in the environment and minimize economic losses and damage to natural systems [1]. Specifically, 90.2% of the world’s plastics production was fossil-based, and post-consumer recycled and bio-based plastics accounted for only 8.3% and 1.5%, respectively. Therefore, several strategies and regulations at European and global levels have been adopted to increase plastic recycling. For example, the new European Strategy on Plastics dictates that, by 2030, all packaging must be reusable, recyclable or compostable. In addition, Directive 2018/852 establishes a gradual increase in recycling rates, reaching 70% in 2030, with a target of 55% for plastic packaging. In this regard, valuing post-consumer plastics and avoiding down-cycling (transforming a material into another of a lower quality than the initial one) are key goals toward a circular economy for plastics.

Mechanical recycling is one of the most attractive strategies to recover plastics after consumption. Polypropylene (PP) is one of the most demanded plastics that can be mechanically recycled and is widely used for packing snacks, pasta, baked goods, rice and beverages. PP has low specific weight, thermal and impact resistance, low cost and is processable through conventional industrial techniques, such as extrusion and injection molding [2]. These properties make PP hardly replaceable by other materials [3]. Nonetheless, PP and other polyolefins degrade during the packaging’s service life and the thermal–mechanical cycles applied during recycling. The occurrence and type of degradation reactions during recycling depend on the chemical structure and grade of the polymer, processing conditions and number of applied reprocessing cycles [4,5,6]. PP recycling leads to processability changes due to modification of viscosity and physical properties of polymers. Most studies have used recycled PP from simulated reprocessing at a laboratory scale and few of them post-consumer rigid PP, and some divergences in the properties’ variations in recycled PP have been found [4,7,8,9]. Post-consumer recycled polypropylene (PCPP) has been less studied due to its complexity in terms of composition and presence of polymer chains with oxidized groups, post-consumer flexible PP even less because of its light weight, which makes it challenging to collect and process. Deterioration of PCPP properties can be associated with two factors: (i) oxidative reactions under mechanical stress and high temperatures that mainly lead to chain scission, but also crosslinking and branching, which modify molar mass and viscosity of a polymer; and (ii) contamination with other polymers of similar specific gravities during recollection [10]. For this reason, use of PCPP is limited and the interest in finding strategies to counteract the negative effect of recycling has increased enormously in recent years.

Nanotechnology is a current alternative to improve the physical–rheological properties of recycled polymers by incorporating low concentrations of nanoparticles in the polymeric matrix. Development of nanocomposites based on recycled polymers has high added value and attractiveness for the polymer and composites industries. Nanoreinforcements promote a large interface surface area that leads to quantitative improvement in the polymer’s performance [11]. The nanocomposite’s resulting properties will depend on the type of polymer and nanofiller, processing conditions, concentration and dispersion of the nanofiller, among others [12,13]. In this context, PP nanocomposites mainly based on nanoclays, calcium carbonate and carbon nanotubes have been developed, focused on thermal–mechanical properties [14]. Nonetheless, fumed nanosilica (NS) has been used to a lesser extent and has only been incorporated in some virgin polyolefins. According to our review, NS incorporation in PCPP, which is a more structurally complex polymer system, has not been reported. Nanocomposites based on NS have been prepared only using virgin PP [15,16]. Thus, further research is required towards a better understanding of the role of NS in PCPP‘s performance.

Properties of nanocomposites depend on the type of polymer, the polymer’s molecular weight and grade, chemical structure, type and concentration of nanofiller, processing conditions and plastic conversion techniques, among others [11]. Studies on nanocomposites with NS have used virgin isotactic polypropylene processed by in situ polymerization, melt mixing, compression molding and hot-drawing, which are not the conventional industrial techniques for obtaining flexible packaging film, which is one of the mean applications of PP, and some differences in property variations have been found [15,16,17,18,19].

Fumed silica is an outstanding alternative because of its commercial accessibility, low cost and uses as a food additive [20]. Fumed silica is an amorphous synthetic silica that is white, odorless and non-toxic and exhibits high surface energy because of its extremely high surface area per mass unit and possesses silanol groups that can be chemically modified by alkylchlorosilanes or alkylsilazanes to provide hydrophobicity [15,21]. Several types of silica nanoparticles can increase strength and stiffness, change deformation and improve the thermal stability of the polymer matrix. However, the reinforcement behavior and role of NS as a decelerator of thermal degradation of some virgin polymers has been previously hypothesized, but further investigation support for PCPP and using the cast-extrusion technique for a more realistic approach is needed to advance in finding alternatives to increase the recycling rates of this post-consumer plastic [22,23].

Thus, in this work, nanocomposites based on post-consumer flexible PP and two types of fumed silica (hydrophilic and hydrophobic) were developed, and their structural, thermal and processability properties were investigated.

## 2. Materials and Methods

### 2.1. Materials

Homopolymer grade virgin polypropylene in pellets (VPP) (MFI = 3.0 g 10 min^−1^ at 230 °C and 2.16 kg) was supplied by Petroquim S.A. (Chile). Post-consumer recycled polypropylene pellets (PCPP) from flexible packages were purchased from Inproplas S.A (Chile). Two types of commercial fumed nanosilica (NS) purchased from Haochuang Material (native particle size ranges from 5 nm to 40 nm) were used: (i) hydrophilic nanosilica (NS1) with a specific surface area of 200 m^2^ g^−1^ and (ii) hydrophobic nanosilica (NS2) obtained after chemical post-treatment of NS with dimethyldichlorosilane.

### 2.2. Preparation of PCPP/NS Nanocomposites

Films and pellets nanocomposites based on PCPP and NS were obtained through extrusion. NS concentrations of 0.5, 1, 2 and 4 wt% with respect to the total mass of the nanocomposite were used, and films were designed as PCPP-XNSY, where X is the weight concentration of the corresponding fumed silica NSY, hydrophilic (NS1) or hydrophobic (NS2). Control films and pellets of PCPP and VPP were also prepared. VPP was used as a reference of a type of polypropylene used in the manufacture of bioriented extruded or coextruded films.

Preparation of films and pellets was carried out by using a twin-screw extruder Labtech Scientific LTE-20–40 (Samutprakarn, Thailand) with a temperature profile from 180 °C to 195 °C from feeding to the extruder die. The screw speed was 35 rpm and the torque was between 40% and 50%. Previously, polymers and NS were dried at 100 °C for 24 h. Thickness of the films was measured by using digital micrometer Digimatic Mitutoyo ID-C112 (Kawasaki, Japan), resulting between 150 and 180 µm. For pellets production, filaments were formed in a round nozzle die and subsequently solidified in a Scientific model LW-100 water bath (Bangkok, Thailand). The filaments were cut at a speed of 10 m min^−1^ in a pelletizer Scientific, LZ-120 (Bangkok, Thailand) coupled to the extruder.

### 2.3. Characterization of the Nanocomposites

#### 2.3.1. Fourier Transform Infrared Spectroscopy (ATR-FTIR)

PP, NS and nanocomposites were analyzed in an FTIR equipment Bruker Alpha IFS 66V (Ettilingen, Germany) coupled to a crystal diamond of attenuated total reflection Bruker Platinum. FTIR spectra were obtained in attenuated total reflectance (ATR) mode in a wavenumber range from 4000 to 400 cm^−1^ with a resolution of 4 cm^−1^ and 64 scans. The spectra analyses were performed with the program Opus v. 7.0.

#### 2.3.2. Thermogravimetric Analysis (TGA)

Thermal stability and degradation temperatures of PP, NS and nanocomposites were evaluated through thermogravimetric analysis with a TGA/DSC 1 analyzer (Schwarzenbach, Switzerland). 6 to 7 mg of each sample (film or NS) were collocated in alumina capsules and heated from 30 °C to 700 °C at 10 °C min^−1^ heating rate under nitrogen atmosphere with a flow rate 50 mL min^−1^. Decomposition initiation temperature at 2.5% mass loss (T_onset_), temperature at the maximum degradation rate (T_d_) and weight percentage of residues at 600 °C were determined.

#### 2.3.3. Differential Scanning Calorimetry (DSC)

The effect of incorporating NS in the thermal properties of the PCPP was analyzed through differential scanning calorimetry (DSC) using a Mettler DSC-822e analyzer (Schwarzenbach, Switzerland). 5 to 6 mg of each film was weighed into aluminum capsules and subjected to three thermal programs: (i) 0 °C to 250 °C (first heating), (ii) 250 °C to 0 °C (cooling) and 0 °C to 250 °C (second heating), with a heating/cooling rate at 10 °C min^−1^ under a nitrogen atmosphere. Melting temperature (T_m_), crystallization temperature (T_c_) and melting (ΔH_m_) and crystallization (ΔH_c_) enthalpies were determined. Furthermore, the crystallinity of the samples was calculated through Equation (1):

X_c_ = ΔH_m_/(ΔH_100_ × X_PP_) (1)

where ΔH_100_ is the melting enthalpy of a whole crystalline polypropylene (207 J g^−1^) [16] and X_PP_ is the mass fraction of the polymer in the sample. DSC analyses were carried out in duplicate.

#### 2.3.4. Rheological Analysis

VPP, PCPP and nanocomposites with 1, 2 and 4 wt% of each NS were analyzed to establish comparisons between nanocomposites with improved thermal stability at equivalent concentrations of NS1 and NS2. Plates of 25 mm × 1.5 mm (1.4 g approx.) were injection-molded from the samples in pellets through a machine Haake MiniJetPro Thermo Fisher Scientific. The injection conditions were a conditioning time of 90 s, injection temperature of 220 °C, pressure of 500 bar, injection time of 5 s, post-injection pressure and time of 200 bar and 5 s and a molding temperature of 60 °C. The plates were subjected to rheological tests in a parallel plate rheometer Anton Paar MCR301 to measure the complex viscosity (η*), storage modulus (G′) and loss modulus (G″) versus frequency (ω) at 190 °C under nitrogen atmosphere in dynamic mode. The frequency range was between 0.1 and 500 rad s^−1,^ which was previously verified to be in the linear viscoelastic range by amplitude sweep tests for polypropylene.

#### 2.3.5. Melt Flow Index

The melt flow index of the PCPP and nanocomposites with 1, 2 and 4 wt% of NS was evaluated to establish comparisons between nanocomposites with improved thermal stability at equivalent concentrations of NS1 and NS2. The tests were carried out in a plastometer Zwick Roell Mflow (Ulm, Germany) following the ASTM D1238-20 standard. 4 to 5 g of sample in pellets were put into the plastometer’s cylinder and heated at 230 °C with an applied weight of 2.16 kg. Six measurements for each sample were carried out with a preheating time of 7 min, and the average value and standard deviation were reported.

#### 2.3.6. Statistical Analysis

The results obtained for DSC and MFI were statistically analyzed through variance analysis (ANOVA) and LSD Fischer’s multiple range test in order to find statistically significant differences between the samples for a random experimental design (*p* < 0.05).

## 3. Results and Discussion

### 3.1. Attenuated Total Reflectance-Fourier Transform Infrared Spectroscopy (ATR-FTIR)

**Figure 1:**
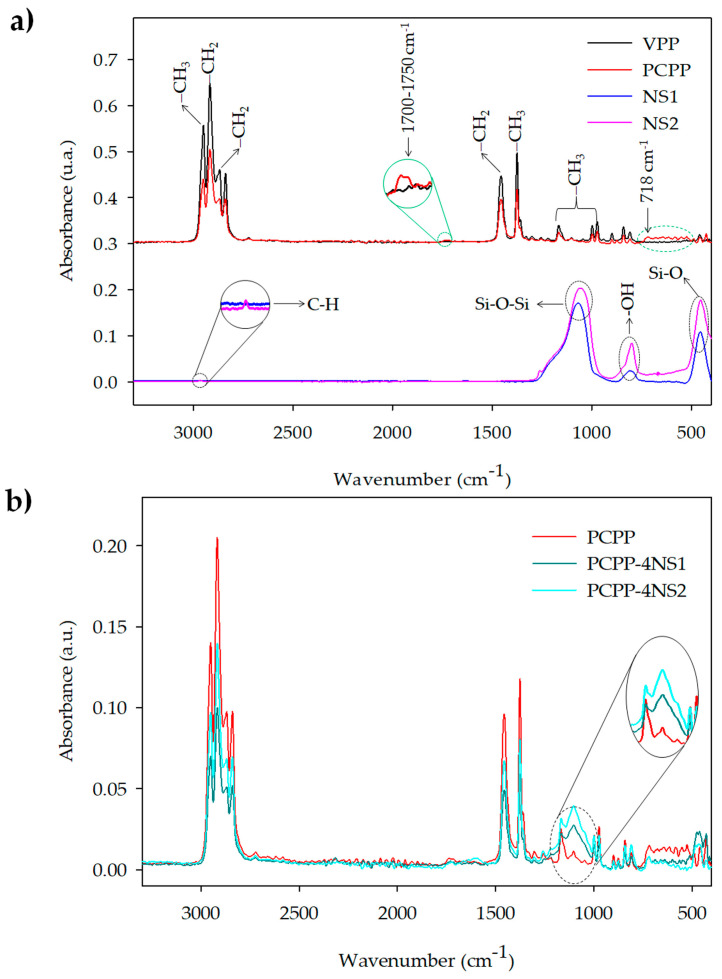
ATR-FTIR spectra of (**a**) VPP, PCPP, NS1 and NS2 and (**b**) representative developed nanocomposites.

Figure 1 shows the ATR-FTIR spectra of NS, control films and nanocomposites with 4 wt% of NS. As Figure 1a shows, the spectra of VPP and PCPP films showed characteristic peaks of PP. Symmetric stretching of –CH_3_ was identified at 2950 cm^−1^. The peaks at 2917, 2838 and 1456 cm^−1^ were attributed to asymmetric stretching, symmetric stretching and symmetric bending vibration of –CH_2_–, respectively. Symmetric bending vibration of –CH_3_ was detected at 1375 cm^−1^, and the rocking vibration of this group was shown at 1167, 998 and 973 cm^−1^. Furthermore, stretching of C–CH_3_ was identified at 840 cm^−1^ [24]. Figure 1a also shows a difference between the VPP and PCPP films in the range of 800 to 500 cm^−1^. The peak identified at 718 cm^−1^ in the post-consumer recycled polypropylene was associated with bending vibration of –CH_2_ of polyethylene (PE) chains [25,26]. This result demonstrated that PCPP contained traces or a minimum proportion of PE, which might have been low-density polyethylene (LDPE), linear low-density polyethylene (LLDPE) or a blend of them. Presence of PE in PCPP is reasonable since recycled polypropylene is from flexible materials, and these polyolefins are typically mixed in collection and recycling processes. Furthermore, a slight increase in the intensity of the peaks between 1700 and 1750 cm^−1^ would be associated with formation of carbonyl groups. For instance, a study developed by Fasihah et al. (2017) associated peaks at 1718 and 1741 cm^−1^ identified in the recycled PP to formation of ketone and ester groups, respectively [27]. Presence of these groups can be a signal of degradation of polymeric chains due to thermo-oxidative stress or exposure to UV radiation [28].

On the other hand, the spectra of NS show three wide bands associated with the characteristic peaks of fumed silica. Symmetric stretching of Si–O–Si was detected at 1067 cm^−1^, bending vibration of –OH in the silanol group at 800 cm^−1^ and rocking vibration of Si-O group at 454 cm^−1^ [29,30,31]. Furthermore, a peak at 2990 cm^−1^ in the NS2 was observed and attributed to the asymmetrical stretching vibration of C–H associated with the organic modifier of the NS (Figure 1a) [32].

Figure 1 shows that incorporation of NS in the PCPP did not produce new absorption bands, suggesting low interaction between the nanoparticles and the polymer. However, presence of NS in nanocomposites PCPP-4NS1 and PCPP-4NS2 increased the intensity of the peak associated with symmetric stretching Si–O–Si (1067 cm^−1^); however, it overlapped with the peak of PCPP related to rocking vibration of –CH_3_ group.

### 3.2. Thermal Stability of the Nanocomposites

**Figure 2:**
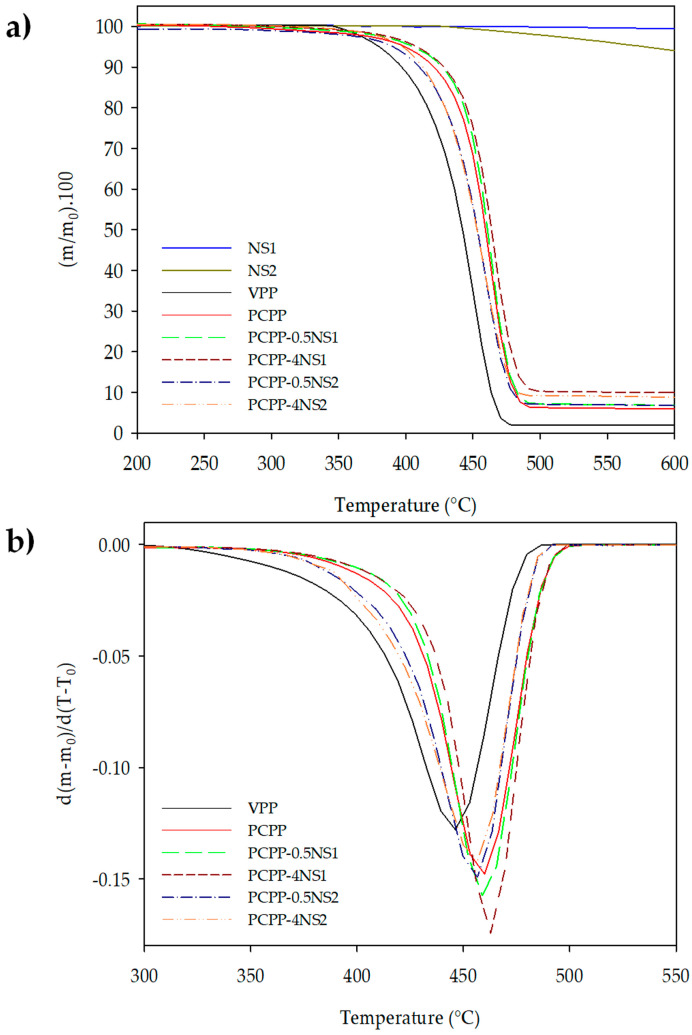
(**a**) TGA and (**b**) DTGA curves of NS1, NS2, control and representative nanocomposite films at 0.5 and 4 wt% of NS.

Figure 2 shows TGA and DTGA curves of NS, control films and the nanocomposites at 0.5 and 4 wt% of NS as representative samples. T_onset_, T_d_ and residue mass at 600 °C of the samples are reported in Table 1.

**Figure 3:**
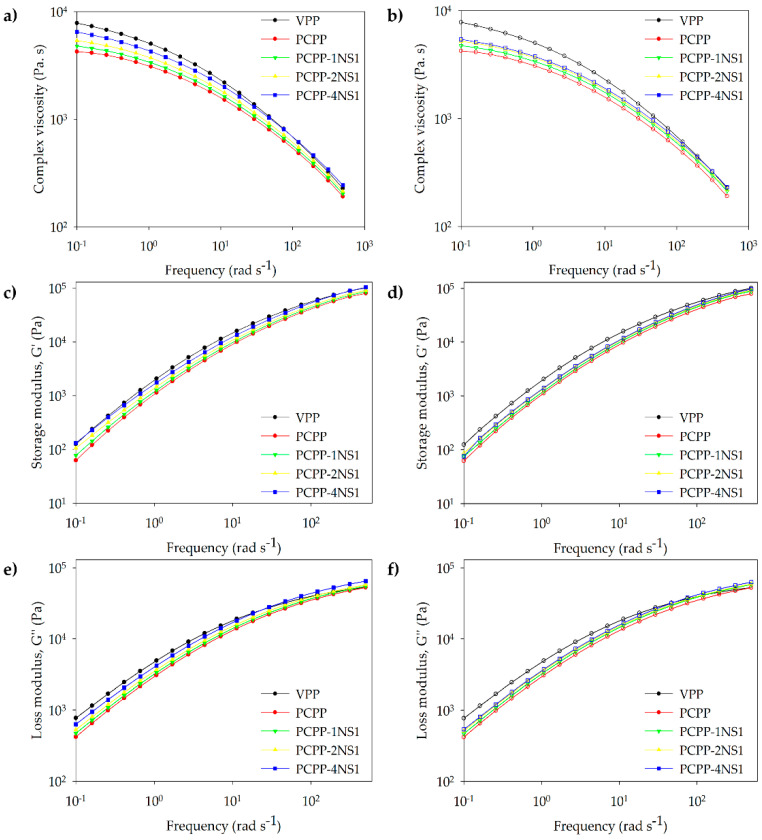
(**a**,**b**) Complex viscosity (η*), (**c**,**d**) storage modulus (G′) and (**e**,**f**) loss modulus (G″) of the samples as a function of angular frequency.

As Figure 2a shows, NS1 was thermally stable in the whole temperature range, while NS2 slightly lost mass from 450 °C, reaching 6 wt% at 600 °C. This result would be associated with partial degradation of the organic modifier of NS2. Similarly, previous studies have not reported mass loss during TGA analysis of non-treated fumed silica under a nitrogen atmosphere up to 950 °C [33,34]. On the other hand, as shown in Figure 3b, VPP film exhibited thermal stability lower than PCPP film and nanocomposites. Presence of PE can explain higher values of T*_onset_* and T*_d_* of PCPP in accordance with ATR-FTIR analysis. PE is more thermally stable and is degraded from 400 °C, as reported by Dikobe and Luyt (2010) in their comparative study of LLDPE and PP and their composites with wood powder [35]. The lower thermal stability of pure PP than PE is explained by presence of methyl group substituents in their polymeric chains that favor formation of free radicals and, thus, its thermal decomposition [36].

On the other hand, as Figure 2b shows, the high thermal stability of NS1 produced an increase in the thermal stability of PCPP, reflected as higher values of T_onset_ and T_d_ of the nanocomposites. In this context, T_onset_ of the PCPP was increased 10 °C when 0.5 wt% of NS1 was incorporated, and 14 °C for nanocomposite PCPP-4NS1. T_d_ of the PCPP with a value of 462 °C was also slightly increased to 465 °C when 4 wt% of NS1 was added. The excellent thermal resistance of NS1 to high temperatures delayed degradation of the polymeric matrix. Furthermore, thermal degradation of the polymer possibly favored agglomeration of NS aggregates on the surface of the melted material, which created a physical barrier to heat in the polymer [37].

Incorporation of NS2 also delayed thermal degradation of PCPP at 1% of NS2 or higher concentrations. Nonetheless, Table 1 shows that nanocomposite PCPP-0.5NS2 exhibited advancement of 10 °C and 5 °C in T_onset_ and T_d_ values compared to the control PCPP film. Furthermore, the T_onset_ of PCPP-0.5NS2 was lower than the T_onset_ of the VPP film. This effect could result from the lower thermal stability of the hydrophobic NS2 and the possible interactions of the organic modifier of the NS2 with the polymer matrix, which favored early degradation of the PCPP. However, an increase in NS2 concentration improved the thermal stability of the nanocomposites, and PCPP-1NS2 was the most thermally stable nanocomposite. This result would be associated with an adequate balance between the concentration and dispersion of the NS in this nanocomposite. However, the highest concentration of NS2 (4 wt%) diminished T_onset,_ possibly due to a greater concentration of organic modifier interacting with the PCPP, and then the effect of better heat transfer to the polymer matrix prevailed and favored its degradation.

On the other hand, PCPP film exhibited higher residue concentration after its pyrolysis than VPP film. Pyrolysis of the VPP resulted in 2% residues and was increased to 6% for PCPP. Furthermore, incorporation and increase in NS produced a rise in residual percentage in the nanocomposites. This result would be associated with inorganic contamination of particles and additives incorporated during recycling of the PCPP, whose amount might have varied among the samples due to the heterogeinity of the recycled plastic used for each extrusion load and the presence of residual NS not degraded during the thermal analysis [38]. Finally, the results showed that there was a trend to higher thermal stability of PCPP when NS1, a nanofiller with higher thermal resistance, was used compared to nanocomposites with NS2 at the same concentration. This effect was more clearly observed for T_d_ values at 2% y 4 wt% of each NS. It is important to highlight that the T_onset_ values of all nanocomposites were below the extrusion temperature of the PP.

### 3.3. Differential Scanning Calorimetry (DSC)

Table 2 shows the DSC parameters and crystallinity of the films. In the first heating, homopolymer VPP film showed a unique endothermic process at 164 °C, associated with the melting of the polymer. On the other hand, two endothermic processes at 124 °C and 164 °C were evidenced in the PCPP film. In this case, the presence of chains of PE detected by ATR-FTIR analysis (Figure 1a), which might be LDPE, LLDPE, medium-density polyethylene (MDPE) or their blends, first produced a melting transition in the recycled polymer. As was mentioned before, this result is a typical finding in recycled polyolefins, where it is common to find blends of PE and PP [39,40]. In the stages of collection and separation during PP recycling, traces of PE generally remain due to their similar specific gravities [9]. A study carried out by Hansen et al. (2019) also evidenced two melting processes in recycled post-consumer polypropylene, and this result was associated with PE traces. The researchers mentioned that PE could come from buckets, bowls and dumps collected while recycling the material [41].

On the other hand, PCPP was less crystalline than VPP, possibly associated with two factors: (i) the commercial VPP was an isotactic homopolymer and (ii) the presence of traces of polymers structurally different from the PP in the PCPP that impeded the ordering of polymeric chains during the cooling for the film production by extrusion. Further, PP reprocessing could generate shorter polymeric chains that favor a lower amount of ordered sites or smaller crystallites whose energy to be melted is lower, as Table 2 shows.

Nanocomposite films had thermal behavior similar to the PCPP control film. This effect was evidenced since their melting temperatures and enthalpies had similar values (Table 2). However, both NS in the nanocomposites slightly favored crystallization of the polymer. This result would be related to the nucleation action exerted by the NS during processing of the nanocomposites, favoring a higher formation of ordered structures during cooling of the PCPP. Furthermore, this nucleation effect also generated different crystalline structures in nanocomposites PCPP-0.5N1 and PCPP-4NS1 that melted between 160 and 164 °C. Dorigato and Pegoretti (2013) also obtained films of virgin PP with higher crystallinity when silica nanoparticles were added. In this study, the authors mentioned that the nucleation action of the nanoparticles enabled obtaining more crystalline materials [18].

Table 2 also shows that cooling of VPP film produced a unique exothermic transition at 114 °C, associated with crystallization of PP. On the contrary, PCPP film exhibited two exothermic transitions at 107 and 113 °C, related to crystallization of PE and PP, respectively. Presence of traces of LDPE, LLDPE or MDPE in the PCPP produced the first thermal transition at the lowest T_c_, whose value is similar to the reported values in previous studies for PE [9,40,42]. Furthermore, there was not clearly observed a trend in variation in released heat during the crystallization regarding type and concentration of NS. This effect could be attributed to the typical heterogeneous composition in post-consumer recycled polymers.

Finally, in the second heating, where the thermal history of the polymer was erased, temperature values and similar thermal behavior to the first heating were registered. However, formation of a unique crystalline structure in the PP with melting temperatures of PP and PE slightly lower than those registered in the first heating process is highlighted. Further, NS addition did not significantly affect such temperature values., and, as in the first heating process, a significant increase in the crystallinity of PCPP by the presence of NS was observed. This fact confirmed the nucleation effect of nanoparticles during manufacture of nanocomposites. Nonetheless, statistical differences in the crystallinity of the nanocomposites were not obtained through variation in type and concentration of NS.

### 3.4. Rheological Analysis

Figure 3a,b shows that PP controls and nanocomposites had the typical non-Newtonian behavior of the polymers in the studied frequency range in accordance with that reported for virgin homopolymer PP (MFI = 2.2 g 10 min^−1^) [43]. The zero-shear rate viscosity of the samples is reported in Table 3. The NS loading increased the zero-shear rate viscosity of the PCPP, resulting in values closer than those of the commercial VPP despite structural and compositional differences between these polymers.

NS type did not influence viscosity values at 1 and 2 wt% of NS, but a significant difference at 4 wt% of NS was found (Table 3). This fact could be associated with the greater interactions between NS and PCPP at the highest NS content. Nanocomposite PCPP-4NS1 had a viscosity 1.19 times higher than the viscosity of PCPP-4NS2, which can be attributed to hydrogen bond interactions between carbonyl groups in the PCPP and OH– groups of the non-treated NS1. In accordance, an increase in complex viscosity with NS concentration in the whole frequency range was observed, which indicated an improvement in the viscoelastic properties of the PCPP (Figure 3a,b). This increase in the viscosity of the nanocomposites associated with the interactions of the oxidized groups in the recycled plastic was interesting considering that, on the contrary, a slight reduction in the complex viscosity of compression-molded virgin PP has been recently reported through adding 1 and 2 wt% of nanosilica treated with hexamethyldisilazane [16].

The storage modulus (G′) and loss modulus (G″) versus frequency for all materials are shown in Figure 3c–f. Smaller values of G′ were registered for PCPP than those of commercial homopolymer VPP, which can be associated with fewer chain entanglements caused by the shorter degraded chains in the PCPP. It has been reported that G′ and G″ are highly dependent on length and polymeric chain entanglement level [43].

Conversely, NS1- and NS2-filled nanocomposites showed higher G´and G’’ values with respect to the PCPP independent of type of NS. The viscoelastic behavior of the nanocomposites containing NS1 or NS2 was similar. All curves showed the typical melt behavior of the PP, with G′ decreasing with lowering frequency, reported as Maxwellian behavior for the nanocomposites based on virgin PP [44]. Furthermore, increasing NS concentration contributed to stiffening of the PCPP matrix due to interactions between PP and nanoparticles that allow improved stress transfer, which was revealed as higher G′ values, as reported for nanocomposites obtained by reactive blending of virgin iPP and hydrophilic silica [15,45]. The differences in G′ and G″ induced by NS addition were most pronounced at smaller frequencies, and the relative effect was less significant with increasing frequency due to the shear thinning behavior verified in the complex viscosity curves (Figure 3). Thus, NS-NS interactions mainly contributed to G′ and G″ at lower frequencies; meanwhile, interactions between PP chains dominate the fluid dynamics at higher frequencies due to confinement of the NS between PCPP chain decreasing, and, therefore, filler–filler and particle–polymer interactions were diminished. Changes in type of interaction of VPP-based nanocomposites with frequency variation have been reported in nanocomposites prepared by reactive blending [15].

**Figure 4:**
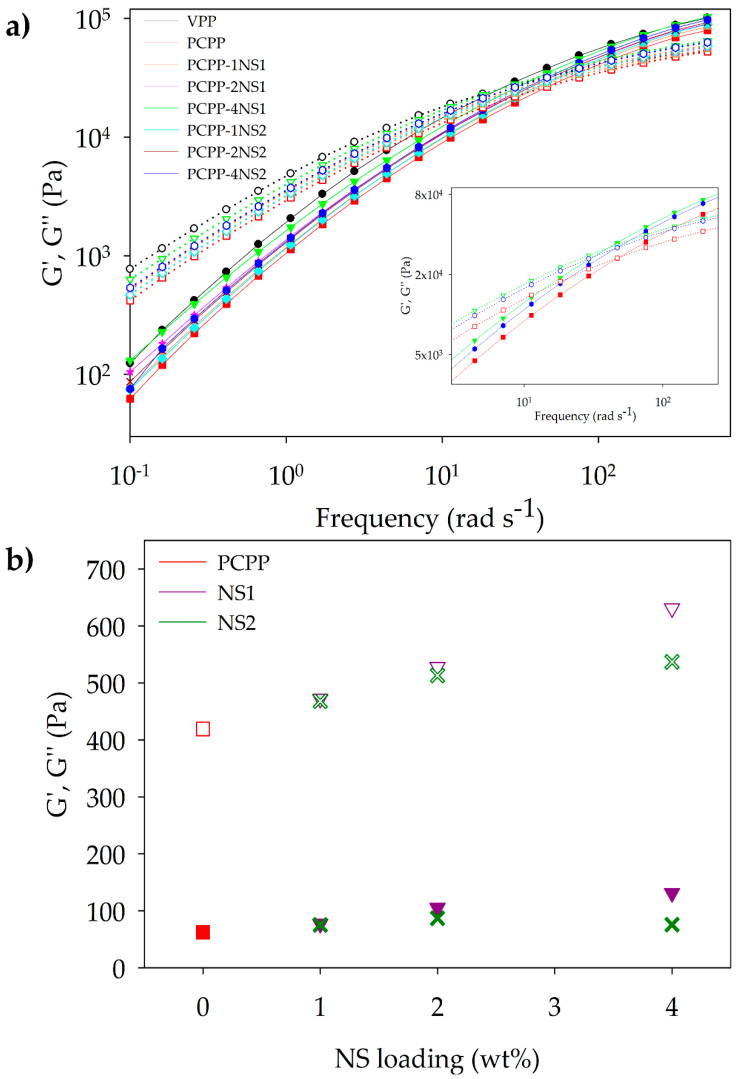
Storage modulus (G′) and loss modulus (G″) of the samples as a function of (**a**) the angular frequency (insert of the intersection of G′ and G″ curves for PCPP, PCPP-4NS1 and PCPP-4NS2 and (**b**) fumed silica loading. G′ and G″ plots of a sample have the same color and marker shape, but filled and empty marks correspond to G′ and G″ modulus, respectively.

On the other hand, Figure 4a shows that the viscous modulus (G″) is larger than the elastic modulus (G′) at low frequency for all samples. The contrary occurred at a crossover frequency, which indicated a transition from viscous/liquid-like to rather elastic/solid-like behavior. This crossover frequency was similar for all samples independent of NS content. Nonetheless, in the insert of Figure 4a, it is observed that 4 wt% of NS1 and NS2 addition caused a slight displacement of the crossover points from 46.9 rad s^−1^ to lower frequency values of 38.6 and 44.7 rad s^−1^, respectively. The transition to a rather elastic/solid-like behavior at a slightly lower frequency for PCPP-4NS1 could be associated with less chain relaxation in this nanocomposite, possibly due to a greater number of more intensive interactions of hydrogen bond types between the hydrophilic NS1 and the oxidized groups in the PCPP. Further, G′ and G″ at 0.1 rad s^−1^ were plotted as a function of NS content in Figure 4b, considering that NS concentration caused viscoelastic properties to be more differentiated at low frequency. In Figure 4b, regarding rheological percolation through NS concentration, it appears that a crossing point of G′ and G″ plots is not observed in the studied concentration range. In this context, a percolation threshold at 4.5 wt% of hydroxyl-functionalized fumed silica (G′ > G″) at a low angular frequency of 0.1 rad s^−1^ has been reported for nanocomposites based on virgin PP prepared via in situ polymerization [15].

### 3.5. Melt Flow Index (MFI)

**Figure 5:**
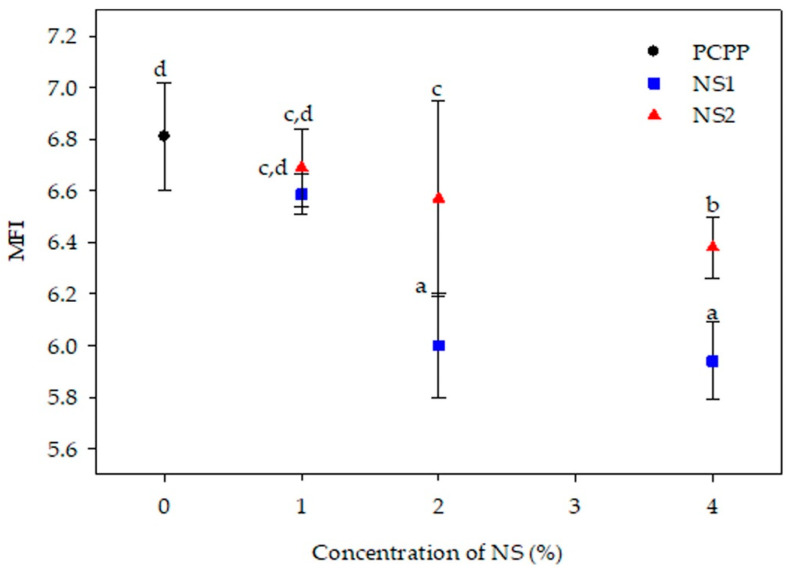
Melt flow index (g 10 min^−1^) of PCPP and developed nanocomposites. Superscripts a–d indicate significant differences in MFI among samples according to ANOVA analysis and LSD Fischer’s test (*p* < 0.05).

MFI measures the capability of a polymer to be adequately processed through techniques with high mechanical stress and pressure as extrusion. MFI is inversely proportional to the viscosity and molar mass of a polymer [5,46]. In this study, the MFI of PCPP and the nanocomposite pellets were determined and are shown in Figure 5.

The MFI of the PCPP was 6.8 g 10 min^−1^, two times higher than the VPP used as a commercial reference (Figure 4). It is important to highlight that several PP types with MFI between 3.5 and 36 g 10 min^−1^ are used in the food packaging industry [47]. Recycling of plastics produces degradation reactions, such as scission of chains, crosslinking and ramification, that modify viscosity and MFI of the polymers. In the case of PP, chain scission and, therefore, a reduction in polymer molar mass, predominates during mechanical recycling. Thus, in the PCPP, the shorter chains reduced the crosslinking and intermolecular interactions and increased the mobility and flow capability of the polymer, expressed as an MFI increase, in accordance with the viscosity reduction observed by rheological analysis [5].

On the other hand, incorporating NS1 at 1 wt% slightly reduced the MFI of the PCPP, and this reduction was more significant at 2 and 4 wt%. High NS1 content favored interactions by hydrogen bonds between the oxidized groups of the PCPP and the silanol groups of the NS1, which increased the viscosity of the nanocomposite. This effect counteracted the negative impact of the degraded recycled polymer on viscosity, which typically tended to be reduced. Moreover, the hydrophilic nature of NS1 enhanced the immiscibility between the nanoparticles and the polymer at the highest concentrations of NS1, causing formation of agglomerates and favoring a drastic reduction in the MFI of the polymer. Previous studies on PCPP composites also evidenced reduction in MFI of polymers by addition of fillers other than nanosilica. Haq and Srivastava (2017) observed a decrease of 1.2-fold for the MFI of recycled PP (9.28 g 10 min^−1^) when 30 wt% wood powder was incorporated [48]. The same effect was observed in a study based on PCPP composites loaded with powder obtained from mollusk shell waste and treated with cashew nutshell and polyethylene glycol. Herein, incorporation of the filler at 20 wt% decreased the MFI of the polymer (8.14 g 10 min^−1^) between 25% and 45%, attributed to a restriction of flowability of the composite with high interaction between the filler and polymer [49].

As with nanocomposites with NS1, incorporation and increase in NS2 concentration in the PCPP reduced its MFI (Figure 5). However, MFI values for NS2-based nanocomposites were higher than those obtained with NS1, evidencing the differences in affinity between the two types of nanosilica and the polymer. The organic modifier of NS2, through its methyl groups, could favor miscibility of NS2-PCPP, but, at the same time, a significantly lower number of hydrogen bond interactions between the hydroxyl groups of the NS and the oxidized groups in the PCPP could be formed. This combined effect resulted in the flowability of the NS2-based nanocomposites being higher than that of the nanocomposites with NS1. Furthermore, lower MFI values for the nanocomposites with respect to pure PCPP indicated that the affinity and interactions between PCPP and NS possibly hindered scission of polymeric chains during extrusion in accordance with the thermal degradation inhibition reported by nanocomposites based on virgin polymer-NS [18]. Thus, lower MFI values for PCPP-NS1 nanocomposites indicated that there was higher degradation hindrance when NS1 was added, and, therefore, higher recovery of viscosity and processability was reached. This degradation hindrance of the PCPP seems to be more significant with NS1 at 4 wt% due to the greater MFI reduction (highest viscosity increase) compared to the control PCPP, reported in Figure 5 and Table 3. These results were consistent with the most significant improvement in thermal stability observed for PCPP-4NS1, which presented both T_onset_ and T_d_ higher than the control PCPP.

## 4. Conclusions

Nanocomposite films based on post-consumer recycled polypropylene and hydrophilic and hydrophobic fumed silica were satisfactorily developed by extrusion. The results indicated that fumed silica hindered thermal degradation, possibly due to interactions between NS and PCPP that hindered PCPP chain scission. PE traces in PCPP and carbonyl groups formed by thermal–mechanical degradation of the polymer during its recycling were detected. Excellent thermal resistance of the NS promoted high thermal stability for PCPP. Hydrophilic nanosilica improved thermal stability for nanocomposites, with 1 to 4 wt% of the filler associated with its thermal resistance and physical barrier to heat. Meanwhile, hydrophobic nanosilica enhanced the thermal stability of the PCPP more significantly at 1 wt% of the nanofiller as a combined result of several influencing factors, such as hydrophobic nanosilica concentration and interactions between the organic modifier and the PCPP, which enabled heat transfer to the polymer. Furthermore, nucleation action exerted by the NS during nanocomposites’ processing slightly favored the polymer’s crystallization.

On the other hand, interestingly, incorporation and increase in NS concentration counteracted the negative impact of the shorter degraded polymer chains in the PCPP. These shorter polymer chains typically cause a reduction in viscosity of a recycled PP, but the interactions of the polymer with the nanosilica, including interactions between the oxidized groups of the PCPP and silanol groups in the nanosilica, increased the complex viscosity more significantly at the highest hydrophilic nanofiller concentration. Thus, PCPP/fumed silica nanocomposites presented improved processability, which appears to be recovery of storage and loss modulus. It is highlighted that rheological performance varied with nanosilica loading, especially at a low frequency range, and was determined through differences in affinity between type of NS and PCPP.

The results on improving thermal stability and viscosity recovery of PCPP by nanosilica addition are promising for potentially increasing the recycling rates of this flexible plastic toward a more circular economy, highlighting that research on the properties of the developed nanocomposites and their reprocessing by industrial plastic conversion techniques for specific applications are needed.

## Figures and Tables

**Table 1:** TGA parameters of the control films and the developed nanocomposites.

Film	T_onset_ (°C)	T_d_ (°C)	Residues at 600 °C (wt%)
VPP	367.1	448.6	2.0
PCPP	372.8	461.8	5.9
PCPP-0.5NS1	382.0	462.5	6.8
PCPP-1NS1	385.5	463.9	7.2
PCPP-2NS1	386.6	464.6	8.0
PCPP-4NS1	387.0	465.0	9.9
PCPP-0.5NS2	363.2	457.1	6.7
PCPP-1NS2	385.4	463.5	7.4
PCPP-2NS2	389.5	455.7	9.6
PCPP-4NS2	380.9	456.7	8.8

**Table 2:** DSC parameters of the control films and the developed nanocomposites.

**First Heating**						
**Film**	**Tm_1_ (°C)**	**ΔHm_1_ (J g^−1^)**	**Tm_2_ (°C)**	**Tm_3_ (°C)**	**ΔHm_2-3_ (J g^−1^)**	**X_c_ (%)**
VPP	-	-	-	164.1 ± 1.6 ^a^	112.4 ± 4.2 ^c^	54.3 ± 2.1 ^c^
PCPP	124.9 ± 1.4 ^a^	4.0 ± 0.4 ^cd^	-	164.3 ± 2.2 ^a^	74.4 ± 3.2 ^a^	36.1 ± 1.7 ^a^
PCPP-0.5NS1	124.2 ± 0.5 ^a^	2.8 ± 0.1 ^a^	160.4 ± 0.2 ^b^	164.4 ± 0.3 ^a^	80.4 ± 0.3 ^b^	39.0 ± 0.2 ^b^
PCPP-1NS1	124.2 ± 0.7 ^a^	2.9 ± 0.2 ^a^	-	163.3 ± 0.8 ^a^	77.3 ± 2.1 ^ab^	37.7 ± 1.0 ^ab^
PCPP-2NS1	125.2 ± 0.1 ^a^	4.4 ± 0.1 ^d^	-	164.2 ± 1.5 ^a^	79.3 ± 2.3 ^ab^	39.1 ± 1.1 ^b^
PCPP-4NS1	124.3 ± 0.7 ^a^	4.2 ± 0.3 ^d^	159.0 ± 1.2 ^a^	164.2 ± 1.1 ^a^	77.1 ± 1.8 ^ab^	38.8 ± 0.9 ^ab^
PCPP-0.5NS2	125.2 ± 0.4 ^a^	3.4 ± 0.1 ^b^	-	164.2 ± 0.1 ^a^	79.1 ± 2.9 ^ab^	38.4 ± 1.4 ^ab^
PCPP-1NS2	125.4 ± 0.5 ^a^	4.4 ± 0.3 ^d^	-	164.6 ± 1.5 ^a^	79.5 ± 3.3 ^ab^	38.8 ± 1.6 ^ab^
PCPP-2NS2	125.1 ± 0.1 ^a^	3.8 ± 0.1 ^bc^	-	164.3 ± 0.7 ^a^	77.9 ± 1.4 ^ab^	38.4 ± 0.7 ^ab^
PCPP-4NS2	125.0 ± 0.1 ^a^	4.1 ± 0.1 ^cd^	-	163.6 ± 1.3 ^a^	77.1 ± 1.6 ^ab^	38.8 ± 0.8 ^ab^
**Cooling**						
**Film**	**Tc_1_ (°C)**		**Tc_2_ (°C)**		**ΔH_c_ (J g^−1^)**	
VPP	-		113.9 ± 0.4 ^abc^		127.0 ± 11.1 ^e^	
PCPP	107.5 ± 0.1 ^abc^		112.8 ± 0.8 ^a^		103.3 ± 3.1 ^a^	
PCPP-0.5NS1	107.3 ± 0.1 ^ab^		113.8 ± 0.1 ^ab^		111.4 ± 0.1 ^abcd^	
PCPP-1NS1	107.0 ± 0.1 ^a^		113.5 ± 0.1 ^ab^		105.8 ± 3.3 ^ab^	
PCPP-2NS1	108.1 ± 0.4 ^c^		115.0 ± 0.5 ^c^		118.2 ± 1.4 ^cde^	
PCPP-4NS1	107.6 ± 0.8 ^abc^		114.1 ± 1.0 ^bc^		106.7 ± 1.6 ^ab^	
PCPP-0.5NS2	108.2 ± 0.1 ^c^		114.2 ± 0.1 ^bc^		114.7 ± 1.3 ^bcd^	
PCPP-1NS2	108.2 ± 0.1 ^c^		114.6 ± 0.5 ^bc^		119.3 ± 6.6 ^de^	
PCPP-2NS2	108.0 ± 0.1 ^bc^		114.2 ± 0.1 ^bc^		111.1 ± 1.2 ^abcd^	
PCPP-4NS2	108.0 ± 0.1 ^bc^		114.5 ± 0.1 ^bc^		109.2 ± 1.7 ^abc^	
**Second Heating**						
**Film**	**Tm_1_ (°C)**	**ΔHm_1_ (J g^−1^)**	**Tm_2_ (°C)**	**Δ** **Hm_2_ (J g^−1^)**	**X_c_ (%)**	
VPP	-	-	161.5 ± 1.3 ^b^	111.2 ± 7.3 ^c^	53.7 ± 3.5 ^c^	
PCPP	123.6 ± 1.3 ^b^	3.2 ± 0.1 ^b^	159.6 ± 2.0 ^a^	73.0 ± 0.6 ^a^	35.3 ± 0.3 ^a^	
PCPP-0.5NS1	121.3 ± 2.4 ^a^	2.8 ± 0.1 ^a^	159.4 ± 0.3 ^a^	82.5 ± 0.6 ^b^	40.1 ± 0.3 ^b^	
PCPP-1NS1	122.9 ± 0.7 ^ab^	2.6 ± 0.1 ^a^	159.1 ± 1.2 ^a^	79.0 ± 1.7 ^ab^	38.5 ± 0.8 ^ab^	
PCPP-2NS1	123.6 ± 0.1 ^b^	4.3 ± 0.1^e^	159.0 ± 0.4 ^a^	82.3 ± 2.0 ^b^	40.6 ± 1.0 ^b^	
PCPP-4NS1	123.3 ± 0.7 ^ab^	4.1 ± 0.1 ^de^	159.1 ± 0.3 ^a^	77.4 ± 0.4 ^ab^	39.0 ± 0.2 ^b^	
PCPP-0.5NS2	123.4 ± 0.1 ^b^	3.6 ± 0.1 ^bc^	158.7 ± 0.1 ^a^	77.1 ± 0.8 ^ab^	37.4 ± 0.4 ^ab^	
PCPP-1NS2	123.8 ± 0.3 ^b^	4.1 ± 0.4 ^de^	159.4 ± 0.2 ^a^	81.3 ± 6.0 ^b^	39.7 ± 3.0 ^b^	
PCPP-2NS2	123.6 ± 0.1 ^b^	4.2 ± 0.1^e^	159.3 ± 0.1 ^a^	78.9 ± 0.8 ^ab^	38.9 ± 0.4 ^b^	
PCPP-4NS2	123.8 ± 0.1 ^b^	3.8 ± 0.3 ^cd^	159.8 ± 0.2 ^ab^	79.4 ± 1.1 ^ab^	40.0 ± 0.5 ^b^	

Superscripts a–e indicate significant differences in the parameters among samples according to ANOVA analysis and LSD Fischer’s test (*p* < 0.05).

**Table 3:** Zero-shear rate viscosity of the samples.

Sample	η_o_ (Pa. s)
VPP	7810
PCPP	4240
PCPP-1NS1	4780
PCPP-2NS1	5380
PCPP-4NS1	6450
PCPP-1NS2	4740
PCPP-2NS2	5210
PCPP-4NS2	5430

## Data Availability

Not applicable.
